# Water spines and networks in G-quadruplex structures

**DOI:** 10.1093/nar/gkaa1177

**Published:** 2020-12-08

**Authors:** Kevin Li, Liliya Yatsunyk, Stephen Neidle

**Affiliations:** Department of Chemistry and Biochemistry, Swarthmore College, Swarthmore, PA 19081, USA; Department of Chemistry and Biochemistry, Swarthmore College, Swarthmore, PA 19081, USA; UCL School of Pharmacy, University College London, London WC1N 1AX, UK

## Abstract

Quadruplex DNAs can fold into a variety of distinct topologies, depending in part on loop types and orientations of individual strands, as shown by high-resolution crystal and NMR structures. Crystal structures also show associated water molecules. We report here on an analysis of the hydration arrangements around selected folded quadruplex DNAs, which has revealed several prominent features that re-occur in related structures. Many of the primary-sphere water molecules are found in the grooves and loop regions of these structures. At least one groove in anti-parallel and hybrid quadruplex structures is long and narrow and contains an extensive spine of linked primary-sphere water molecules. This spine is analogous to but fundamentally distinct from the well-characterized spine observed in the minor groove of A/T-rich duplex DNA, in that every water molecule in the continuous quadruplex spines makes a direct hydrogen bond contact with groove atoms, principally phosphate oxygen atoms lining groove walls and guanine base nitrogen atoms on the groove floor. By contrast, parallel quadruplexes do not have extended grooves, but primary-sphere water molecules still cluster in them and are especially associated with the loops, helping to stabilize loop conformations.

## INTRODUCTION

Water molecules are essential for nucleic acid stability (1–3). This was first demonstrated with fibers of duplex DNA, when hydration was found to be essential for ordered A- and B-DNA diffraction patterns to be obtained (4,5), a key step in the path to the determination of the double-helix structure for DNA (6). Subsequent X-ray crystallographic and NMR studies have revealed discrete associated water molecules in A-, B- and Z- duplex DNA and RNA oligonucleotide sequences (7–16), as well as in their drug and protein complexes (17–19). Patterns of structured water molecules have been found in the narrow A/T minor groove regions of B-DNA crystal structures, defining a stable spine of hydration connecting backbone and base edges (7–16), which serve to stabilize DNA structure, and have to be competed off for drug or protein binding. Clusters of water pentagons and hexagons have been observed in crystal structures of DNA–drug intercalation and minor groove complexes (20–22).

Quadruplex DNA and RNA are higher-order arrangements formed by nucleic acid sequences containing tandem repeats of short G-tracts (23,24). They are widely, though not randomly, distributed in human and other genomes, with over-representation in telomeric regions, in promoter sequences and in untranslated regions. Within the human genome, quadruplex sequences are over-represented in cancer-containing genes (25,26), which has led to them being the focus of much interest as drug targets (27,28).

**Figure 1.**
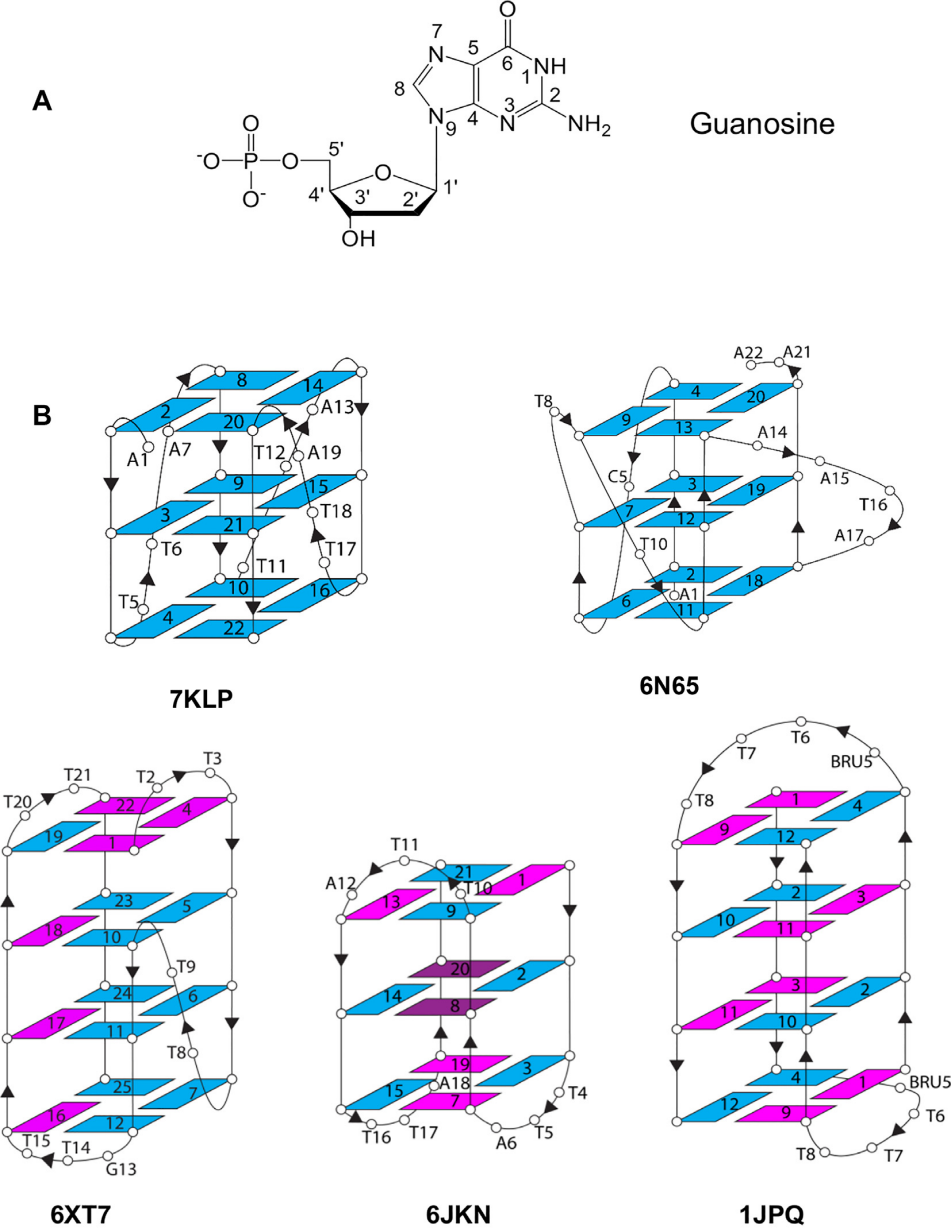
(**A**) Structure of guanosine in an *anti* glycosidic angle conformation, showing the standard nucleotide numbering scheme (50). (**B**) Topology of the five quadruplex crystal structures analyzed in this study. The arrows show the directionality of the phosphodiester strands. Guanosines in an *anti* conformation are colored blue and in the *syn* conformation are magenta. The brominated nucleoside 8Br-dG in structure 6JKN is colored dark magenta and adopts a *syn* conformation. BRU5 corresponds to 5-bromouridine.

Quadruplex structures comprise a core of G-quartets, connected by variable-sequence nucleotide loops. A quartet itself comprises four in-plane guanine bases (Figure 1A) bound by Hoogsteen G:G base pairing, and held together by the sugar-phosphate backbone. In addition, unimolecular and bimolecular quadruplexes invariably contain (normally short) regions of generalized sequence that can form extra-helical loops.

We have surveyed the available folded right-handed quadruplex crystal structures in the Protein Data Bank (29). Almost all report the presence of water molecules, which is unsurprising given the typical water content of at least 40% for an oligonucleotide crystal (30). Examination of these structures reveals fragmented indications of water networks—most of the structures are of insufficient resolution for any firm conclusions to be drawn. However, the availability of several high-resolution crystal structures at greater than 1.6 Å resolution, including two unpublished (but deposited) structures from the Yatsunyk group, has provided us with a resource with which to fully examine the question of whether structured water molecules and networks exist in folded quadruplex structures, and if so where do they form and what is their nature. We report here on an initial study of a set of DNA quadruplex structures (Table 1), which have also been deliberately chosen to have diversity in quadruplex folds. There are insufficient high-resolution RNA quadruplex crystal structures available at present for an analogous study of them to be undertaken.

**Table 1.** Structures analyzed in this study, and their characteristics

PDB id	G4 origin	G4 type	Sequence	Resoln (Å)	R_final_	No. of waters	Groove widths (Å) W/M/N^d^	Ref.
7KLP^a^	Human telomeric	Parallel	AG_3_(T_2_AG_3_)_3_	1.35	0.18	111	M: 16.7 ± 0.4	-
6N65	k-*RAS* promoter	Parallel	AG_2_GCG_2_(TG)_2_G_2_A_2_TAG_3_A_2_	1.60	0.21	64	M: 16.1 ± 0.4	(46)
6XT7^b^	*Tetrahymena thermophila* telomeric	Hybrid	GT_2_(G_4_T_2_)_3_G_4_	1.56	0.17	286	W: 21.1 M: 16.0 ± 0.7 N: 8.6	-
6JKN^c^	Human telomeric	Anti-parallel	G_3_T_2_AGG^Br^GT_2_AG_3_T_2_AGG^Br^G	1.40	0.16	119	W: 20.3 ± 0.6 N: 9.3 ± 0.7	(47)
1JPQ	*Oxytricha nova* telomeric	Bimolecular anti-parallel	(G_4_T_4_G_4_)_2_	1.60	0.24	131	W: 21.5 M: 16.3 ± 0.1 N: 9.1	(38)

^a^Yatsunynk *et al.*, unpublished. Two other human telomeric parallel quadruplex structures are available in the PDB, the original, 1KF1 (48), at 2.1 Å resolution, and a more recent determination, 6IP3 (49) at 1.40 Å resolution.

^b^Yatsunynk *et al.*, unpublished. Four G4s in the asymmetric unit.

^c^The 8Br-dG base is in position 8 and 20.

^d^Grooves W/M/N – Wide/Medium/Narrow; widths of grooves of the same type were averaged and associated error is reported.

## MATERIALS AND METHODS

Crystal structures have been taken from the PDB (Table 1). Water-water and water-DNA hydrogen-bond contacts were systematically explored using a simple algorithmic approach, with a cut-off for a maximum H-bond distance of 3.2 Å. Visualizations and groove width measurements were performed with the programs ChimeraX v 1.1 (https://www.cgl.ucsf.edu/chimerax/) (31) and COOT (v. 0.9.9.1) (https://www2.mrc-lmb.cam.ac.uk/personal/pemsley/coot/) (32). Groove widths were defined as the inter-strand distances between phosphate groups of an individual quartet (measured at the phosphorus atoms). These calculations were run for each P…P distance in a groove and then averaged. Venn Diagrams were created by first extracting water contacts from the .pdb files using program *ncont* in the CCP4 suite (33,34). The extracted data was then used to generate Venn diagrams (https://CRAN.R-project.org/package=venn).

## RESULTS

The high-resolution quadruplex crystal structures selected here represent the common right-handed quadruplex topologies: parallel, mixed hybrid and anti-parallel (Table 1 and Figure 1B). Unlike duplex DNA, which is characterized by two grooves, wide and narrow, quadruplexes have four grooves, each bounded by phosphodiester chains. These grooves have varying widths. In parallel quadruplexes, typified by the human telomeric quadruplex crystal structures, all G-bearing strands are oriented in the same direction and all grooves are of medium width. In the hybrid structures, three out of four G-bearing strands are oriented in the same direction. Hybrid structures have three types of grooves: wide, medium, and narrow. Anti-parallel structures have two G-bearing strands oriented one way and two oriented in the opposite direction (for example up-up-down-down in the 1JPQ structure or up-down-up-down in the 6JKN structure) and two (wide and narrow) or three (wide, medium, narrow) types of grooves.

In general, two adjacent strands running in the same direction will generate a medium groove; and two adjacent strands running in opposite directions will generate either narrow or wide grooves. In a narrow groove, all phosphates point into the groove, thus in the adjacent groove all phosphates on the shared strand between the two grooves necessarily point away from the groove making it either wide or medium but not narrow. In short, two adjacent narrow grooves do not exist. A wide groove has all phosphates pointing away from the groove.

All the structures analyzed here show extensive arrangements of primary-sphere water molecules. Water molecules fill both, the narrow and medium groves in these structures but only the narrow grooves display extended quasi one-dimensional networks of water molecules. These networks by analogy with the water arrangement in the narrow minor groove regions of duplex DNA, are termed ‘spines’.

### The details of quadruplex hydration

#### Parallel unimolecular quadruplexes are associated with small water clusters

**Figure 2.**
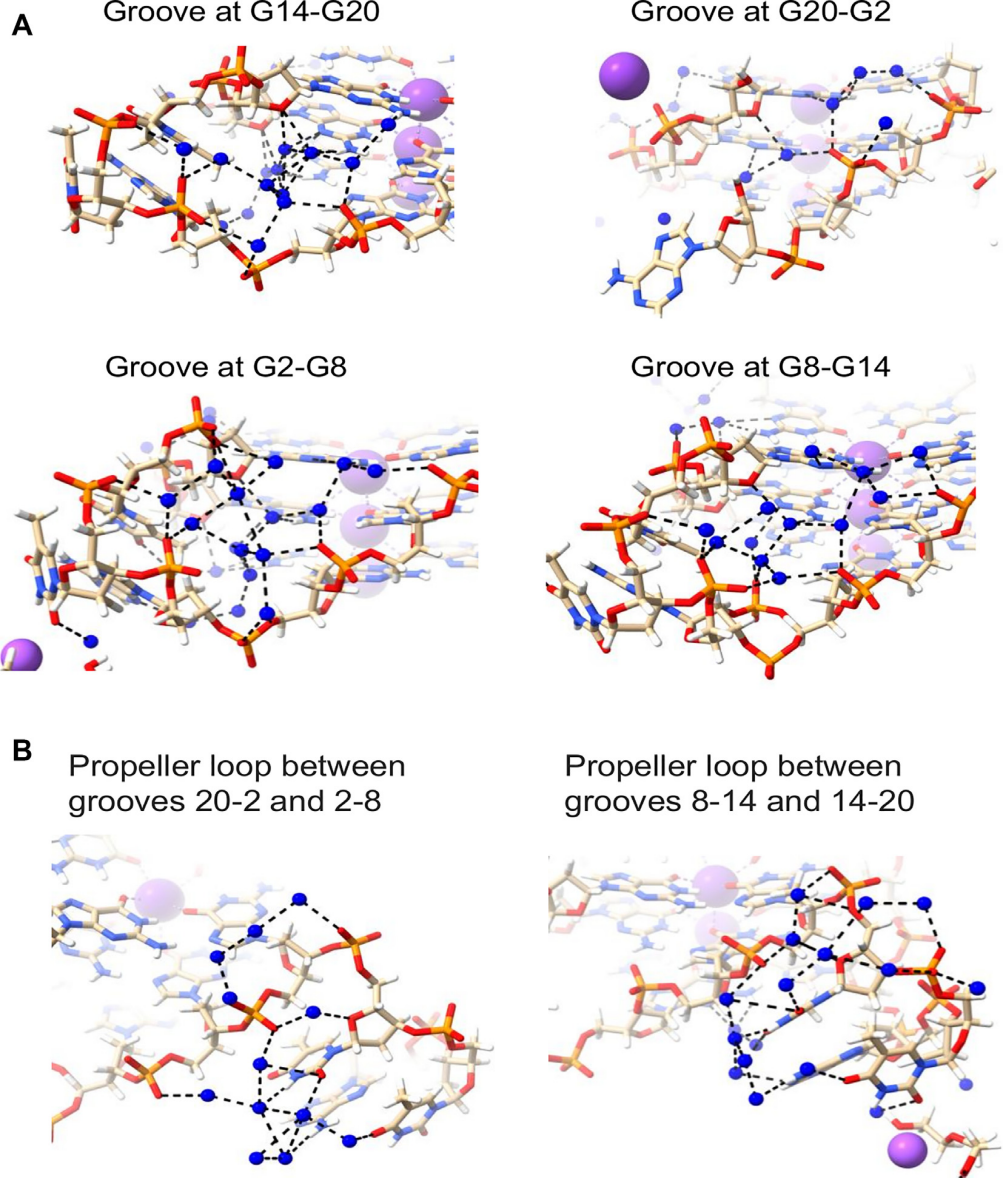
Water clusters and networks in the 7KLP structure. Water molecules are depicted as blue spheres. Potassium ions are shown as mauve spheres. Water networks are depicted in (**A**) all four medium grooves and (**B**) in the T–T–A loops.

Structure 7KLP folds into a three-quartet parallel quadruplex with all grooves being of medium width, 16.7 ± 0.4 Å. Three propeller T–T–A loops extend across three of the grooves. Because of the geometry of Hoogsteen base pairs in the G-quartets, the only non-carbon atoms at the floor of the grooves accessible to hydrogen bonding are N2/N3. Those N2/N3 atoms are always observed to be on the same side of the medium groove, for example the left-hand side in Figure 2A. The guanosines adopt an *anti* glycosidic conformation (Figure 1A) and the O4′ of the deoxyribose sugar is oriented into the groove on the N2/N3 side, and outwards on the C8 side. Interestingly, whether the nucleotide is *anti* or *syn* does not affect the positioning of that nucleotide's phosphate or the O4′ atom relative to its neighbours. The base is simply flipped to account for the *anti* vs *syn* difference (see Supplementary Figure S1).

The water networks within the grooves of structure 7KLP do not have any extended spines of hydration and rely almost exclusively on N2/N3 + O4′ → phosphate water contacts; specifically, the O4′ atoms of the deoxyribose sugars are conveniently positioned for hydrogen bonding via primary-sphere waters with the N2/N3 atoms of guanines from the G-quartet immediately below. These waters then can interact with the secondary-sphere waters, eventually terminating in a hydrogen-bond with a phosphate group on the opposite side of the groove, or one belonging to a loop nucleotide. Waters hydrogen bonded to the guanines from the 5’ terminal G-quartet display only N2/N3 → phosphate connectivity as ‘above’ O4’ is absent. The N2/N3 + O4’ → phosphate water bridges pull the sides of the grooves together. And because the guanosines with a C8 atom in the floor of groove have their N2/N3 side forming the floor of the adjacent groove, these bridges continue across all the grooves, forming in effect a water ribbon wrapping around the quadruplex and further stabilizing the overall structure. These water patterns are depicted in Figure 2A.

The conformations of T–T–A loops in parallel quadruplexes have been found (35) to be maintained by the water networks within and adjacent to the loops. Most phosphates of loop nucleotides point inward and are connected by the dense water clusters (i.e. phosphate → phosphate water contacts). The bases, which point outward, interact with waters through O4′, the O2, N3 and O4 atoms of thymine and N6 of adenine (Figure 2B). The groove defined by G20–G2 is the only groove not obstructed by the propeller loop, therefore, the phosphate → phosphate water connectivity is not observed here.

**Figure 3.**
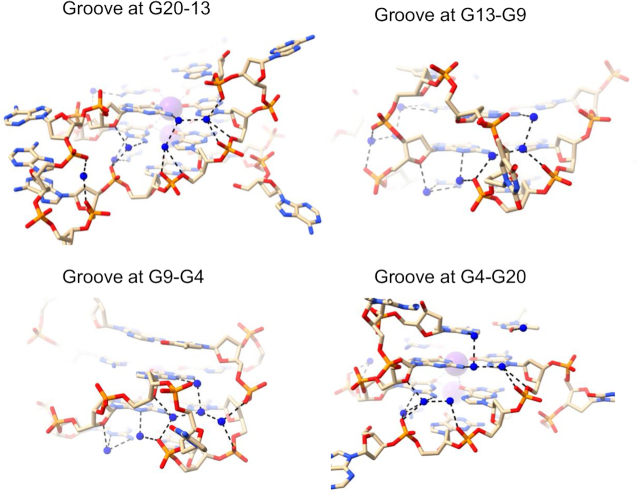
Water networks in all four medium grooves of 6N65 structure. Water molecules are depicted as blue spheres.

Another high-resolution parallel three-quartet quadruplex structure explored in this work is structure 6N65 from the *KRAS* oncogene promoter. As expected, all grooves are of medium width (16.1 ± 0.4 Å) and all the guanosines are in *anti* conformations. There are three propeller loops (C, T and A–A–T–A) and one T bulge. Water networks in 6N65 are similar with those observed in 7KLP, albeit less extensive (Figure 3). The groove at G20-G13 contains a three-water cluster connecting N2 of the top guanine to three different phosphates (N2 → phosphate) followed by two individual water molecules connecting N2 and a deoxyribose sugar O4′ to phosphate (N2 + O4′ → phosphate) and ending with two phosphates connecting to each other (phosphate → phosphate). The groove at G13–G9 has a network of four waters interacting via N2/N3 → phosphate contacts. There are no hydrogen bond interactions involving O4′ in the middle quartet of this groove because the first guanosine adopts a conformation intermediate between *anti* and *syn*, causing the O4′ atom to be oriented away from the groove. The groove at G9-G4 contains a five-water cluster connecting top layer N2/N3 to two phosphates and mid layer N2 to top layer O4′ and a third' phosphate. Finally, the groove at G4–G20 has two three-water clusters. The first cluster connects two N2 to a phosphate, while the second cluster connects O4′ and N2/N3 with two phosphates. There are no interactions between clusters.

#### Hybrid and anti-parallel quadruplex structures have well-characterized water spines

**Figure 4.**
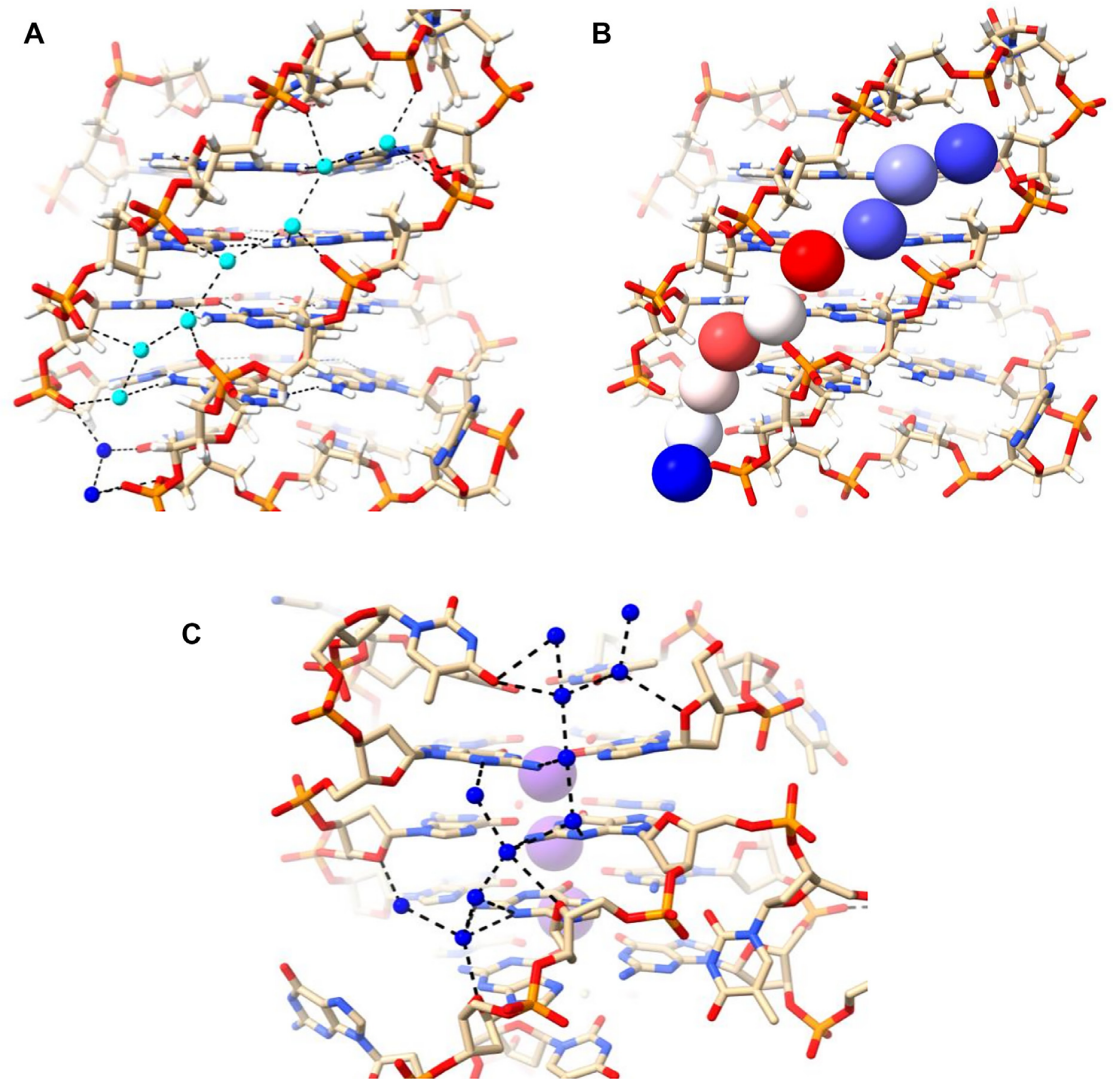
Water networks in a narrow (**A, B**) and wide (**C**) grooves of 6XT7. The narrow groove is that at G19-G22 and the wide groove is that at G1-G19. The T20–T21 loop is at the top right. Deep-spine waters are colored cyan while other waters are colored blue in (A) and are shown as van der Waals spheres and colored according to their crystallographic temperature factors in (B). Red spheres represent the most mobile atoms, with higher *B* values, white spheres represent intermediate mobility and blue have the least mobility. The *B* factors range between 36 and 55 Å^2^.

Structure 6XT7 has four quadruplexes in the crystallographic asymmetric unit. All have identical (3+1) hybrid quadruplex topology (Figure 1B) and nearly equivalent hydration features. We have focused our attention on one representative quadruplex of the four, formed by chain B. This quadruplex has one narrow (8.6 Å width), two medium (16.7 and 15.3 Å widths), and one wide (21.1 Å width) grooves. The narrow groove at G19–G22 has a lateral T20–T21 loop at the top end linking the two strands and is about 22 Å long. The groove is filled by a zig-zag spine of seven connected water molecules, plus two more at the wide bottom end (Figure 4A). All nine waters hydrogen-bond to phosphate groups, and for the most part these interactions alternate between strands. In the center of this water spine there is alternate hydrogen bonding to N2 of a guanine in each successive G-quartet edge. No waters in this grove contact O4′ atoms, either directly or indirectly. The spine water molecules are mostly relatively immobile primary-sphere waters (Figure 4B) and have an average temperature factor <*B*> of 42.4 Å^2^, compared to <*B*> of 39.4 Å^2^ for the DNA atoms. The water network in the wide groove of 6XT7 (Figure 4C) is mostly formed by connections with N2/N3 of guanines as well as connecting to O4' of sugars on both side. This creates a short spine that runs down the middle of the groove, with the wider distance between the phosphate backbones of this groove allows greater accessibility to N2/N3 and results in reduced phosphate interactions.

**Figure 5.**
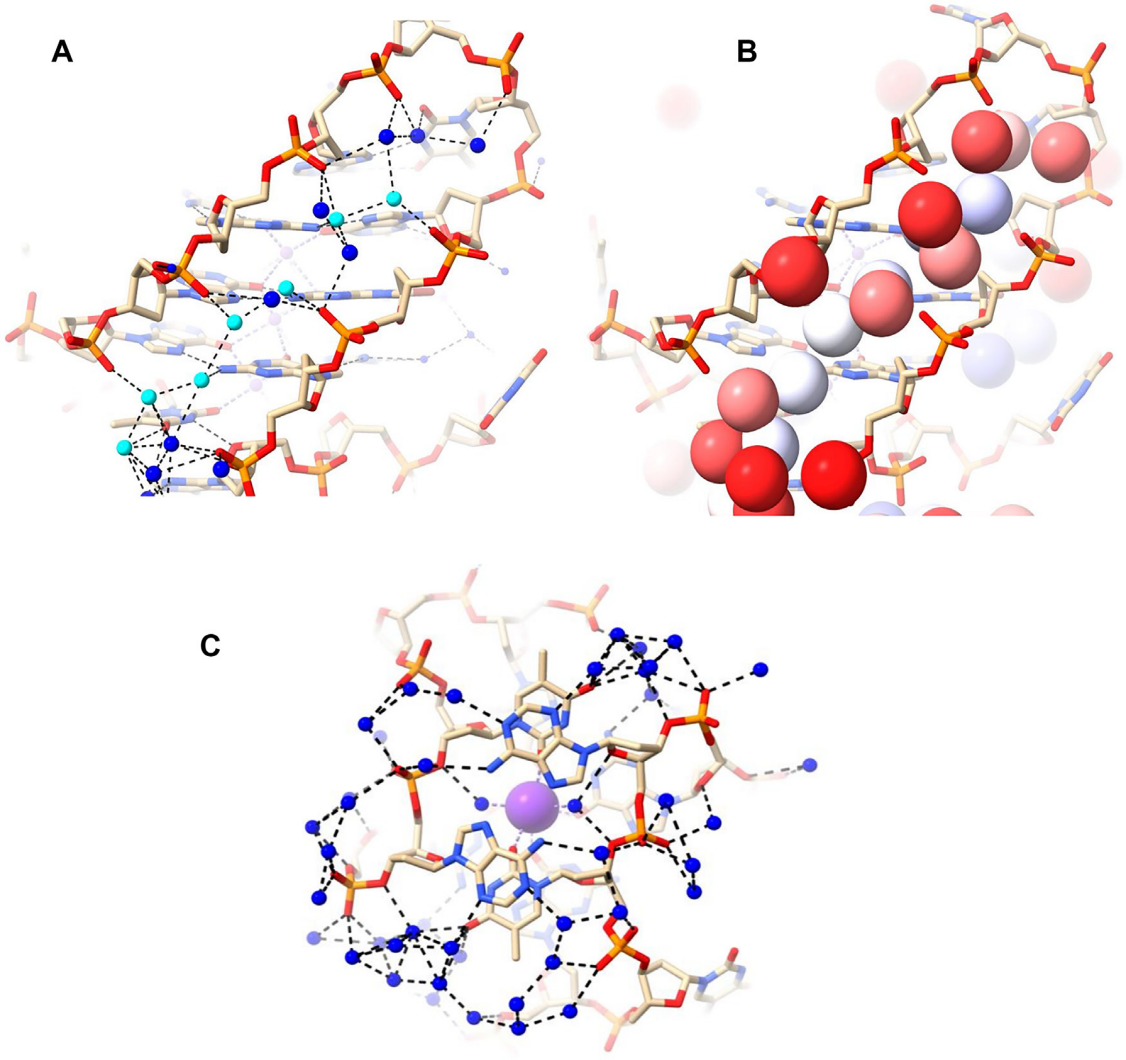
Water network in a narrow groove at G9–G13 of 6JKN. The T10–T11–A12 loop is at the top right in (**A**) and (**B**). Deep-spine waters are colored in cyan while other waters are colored in blue in (A) and colored according to their *B* factors in (B), as in Figure 4B. (**C**) The extensive water network at the base of the structure connecting two narrow grooves (top right and bottom left). A potassium ion is colored purple. The bases in the top layer represent A6 and A18. The water network shown connects the narrow groove networks to each other, pulling the phosphates together throughout the structure. The network here can be considered to comprise three key patterns. The first bridge pattern starts from a phosphate on one side and uses the adenine in the middle to reach the phosphate on the other side. The second pattern starts from the phosphate of one side and eventually connects to the thymine (underneath each adenine), which is connected to the backbone on the other side. The third pattern involves bridges that start and end with phosphates, visible at the bottom of the figure.

Structure 6JKN is a three-quartet intramolecular anti-parallel chair quadruplex with two bromo-substituted guanines in the middle quartet (Figure 1B). 6JKN has two narrow grooves (8.6 and 9.9 Å widths) and two wide grooves (19.7 and 20.8 Å widths). This structure contains three T–T–A lateral loops, two capping wide grooves and one capping the narrow groove. Water molecules form a continuous spine in the long narrow groove at G9–G13 of this structure, which extends from the second T10–T11–A12 loop, along the length of the three-quartet core, to the third T16–T17–A18 loop at the other end, a distance of ca 22 Å (Figure 5A). These water molecules completely fill the groove leaving no space for additional molecules (waters or others). The deoxyribose sugar O4′ atoms are oriented such that they are too distant for hydrogen bonding with groove waters. At the same time, the phosphates point into the groove. The phosphate-to-phosphate groove width is 8.6 Å at G9–G13, narrowing to 8.0 Å at the mid-point of the groove at G8–G14, then widening to 9.3 Å at the lower end at G7–G15, just before the third loop. The spine consists of 18 water molecules, of which 17 are in the primary-sphere, hydrogen bonding to phosphate oxygens, N2/N3 of G-quartet edges, and to O2 of loop Ts. There is one hydrogen bond to an O4′ atom of the adenine at the end of the third T16–T17–A18 loop. This is the only O4’ atom that is oriented towards the groove. Overall, the water spine in the narrow groove of 6JKN closely resembles that in 6XT7. All the phosphates lining the groove are involved in interactions with water molecules, apart from the thymine phosphate at the 5’ end of the second T10–T11–A12 loop, which is pointed away from the groove and is at the upper end of the groove channel. The phosphates have one, two or in two instances, three hydrogen bonds to water molecules. They are at the corners of several water-water triangles, rectangles and pentagons.

The primary-sphere waters in the narrow groove at G9–G13 can be further classified into

- seven waters that are embedded deep in the groove (‘deep spine waters’) and have low temperature factors (Figure 5B; spheres colored according to mobility), with <*B*> of 32.9 Å^2^; these are the same waters colored in cyan in Figure 5A;
- five waters that are close to the edge of the groove (‘mid spine waters’), with <*B*> of 46.8 Å^2^,
- five waters that are at or even beyond the outer edge of the groove (‘outer spine waters’) with <*B*> of 55.2 Å^2^.

The second narrow groove at G21–G1 is shorter as it is not capped by the loop. It contains a rather similar water network to that observed in the G9–G13 groove but the network stops at the top quartet. The two networks in the narrow grooves are connected by an extensive water network as shown in Figure 5C. Here, the terminal G-quartet, G3–G7–G15–G19, π-stacks on two thymines from the two lateral T4–T5–A6 and T16–T17–A18 loops, which in turn stack on two hydrogen bonded adenines, A6 and A18. Thymines and adenines participate in the water network with neighbouring phosphates to maintain the observed secondary DNA structure. The water network in the wide groove is modest in extent and has features similar with those found in structure 6XT7. Despite the availability of O4’ atoms, it relies on N3 atoms, a few N2 contacts, and one phosphate interaction.

**Figure 6.**
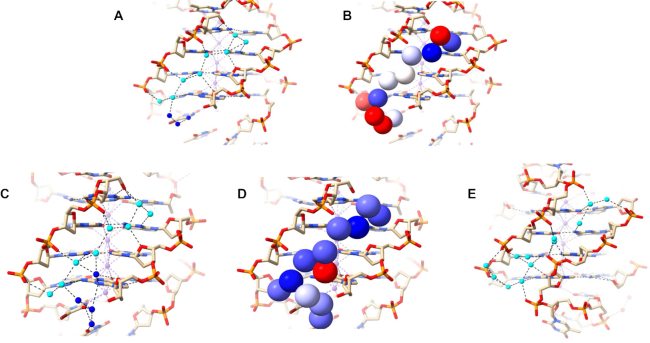
(**A–D**) Water network in the medium grooves of 1JPQ. Deep spine waters are colored in cyan while other waters are colored in blue in (A, C). They are shown in van der Waals representation and colored according to their *B* factors in (B, D), as in Figure 4. (**E**) Water network in the narrow groove of structure 1JPQ.

The 1JPQ structure shows a four-quartet bimolecular anti-parallel G-quadruplex with a diagonal T–T–T–T and a T–T–T–BrU loop. The strands adopt up–up–down–down directionality, unlike the up–down–up–down strand orientation observed in the anti-parallel 6JKN structure. The 1JPQ quadruplex has one wide groove (21.5 Å width), two medium grooves (16.3 Å width each), and one narrow groove (9.1 Å width), all shorter (17, 14, 15 and 13 Å respectively) than the grooves in 6XT7 and 6JKN. The water arrangements in the two medium grooves of 1JPQ are shown in Figure 6A–D. They can be described as short clearly defined spines, albeit slightly more irregular ones than in structures 6XT7 and 6JKN. Linked water molecules contact the phosphate groups on one strand, and O4′ atoms on the other, whilst also contacting N2 and N3 guanine atoms on the groove floor. The eight deep spine waters in the two medium grooves have <*B*> values of 26.8 and 29.1 Å^2^ with values ranging between 21.9 and 32.1 Å^2^. The bases in the top layer represent A6 and A18. The water networkbeing shown connects the narrow groove networks to each other, pulling the phosphates, throughout the structure, together. The network here can be broken into three At first sight the width of these two medium grooves would not be expected to enable even short water spines to exist. However, the consistent inward-pointing orientation of the phosphate groups has enabled these spines to be formed.

The short narrow groove of 1JPQ (13 Å long) contains two groups of connected water molecules (Figure 6E). They do not quite link up to form a continuous network and have more irregularity than the water spines in structures 6XT7 and 6JKN (Figures 4A,B and 5A,B). The discontinuity suggests that one or more water molecules may have not been found in the original structure determination. The waters that are present make hydrogen-bond contacts with phosphate groups and N2/N3 base edges. There are no contacts involving O4’ atoms, which are all oriented away from the groove.

**Figure 7.**
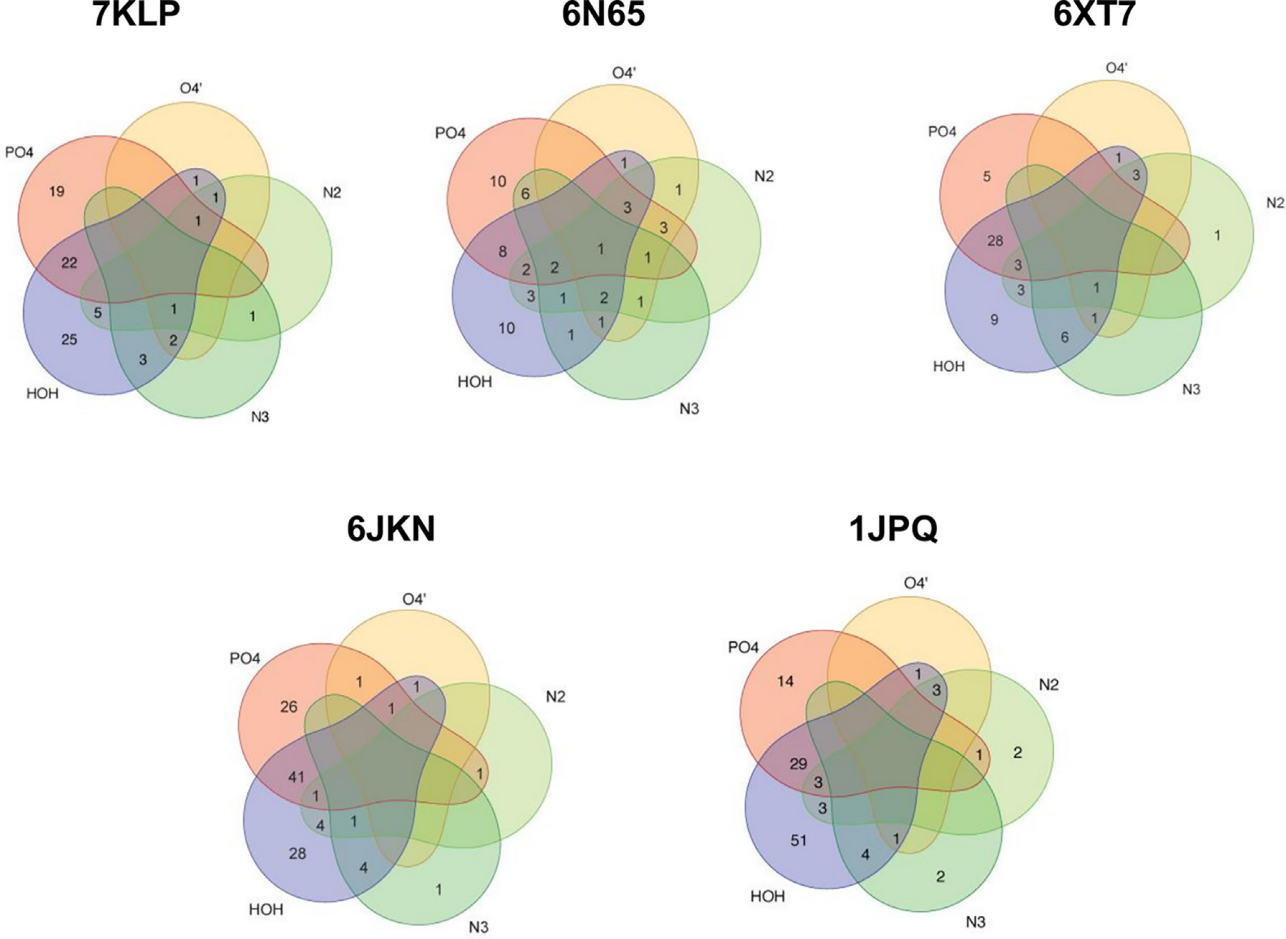
Venn diagrams for the primary-sphere waters in the structures analyzed here. Waters bonded to phosphates are represented by a red area; to sugar O4′ are represented by a yellow area; to N2/N3 of guanines are represented by green areas, and to other waters by purple area. Waters in the non-overlap areas represent bonding to one type of DNA feature or other waters. Waters in the overlap areas represent bonding to multiple DNA features and to other waters.

To summarize the primary-sphere water interactions in all the structures under investigation, we have built Venn diagrams (Figure 7) using the collected numeric data shown in Supplementary Tables S1–S4. Phosphate groups are by far the most frequent of the DNA hydrogen-bonding groups interacting with primary-sphere waters, followed by the N2 of guanine, the N3 of guanine and O4′ of sugar. The most common two-way interactions for the primary-sphere waters are with any of the DNA atoms (phosphates, N2, N3 and O4′) and a secondary-sphere water. Three-way interactions may include phosphate–N2–water; N2/N3–O4′–water; and N2–N3–water. Four-way interactions exist but are rare.

## DISCUSSION

This study has revealed that high-resolution crystal structures of folded quadruplexes have well-defined structured water networks in their grooves and loops, whose nature and extent depends on quadruplex topology. The primary-shell water molecules in these cavities are in hydrogen-bonded contact with (a) phosphate oxygen atoms at the outer edges of the grooves, (b) O4′ sugar atoms at the outer edges of the grooves, and (c) N2/N3 atoms of guanines at the edges of the G-quartets that form the floors of the grooves.

Extended spines of hydration are apparent in the elongated narrow grooves formed in anti-parallel and hybrid structures, but not in the parallel structures examined here, since these cannot form such long grooves. This is undoubtedly due to the invasion of the grooves in the latter structures by fold-back (propeller) loops, which restrict groove lengths. Instead, in folded parallel quadruplexes water clusters are apparent at the loop-groove interfaces, which extend into the loops. Here, the waters appear to play an important role in maintaining particular loop conformations (35). The extended water spines require a groove to be no wider than ca 10.5–11 Å, although as found in the 1JPQ structure, wider medium grooves with inward-facing phosphates can also accommodate water spines, albeit less extended. Wide grooves have fewer structured (i.e. relatively immobile) water molecules, at least as observed in crystal structures.

The most structured water arrangements are the water spines in the narrow grooves of structures 6JKN and 6XT7. The continuous array of water molecules hydrogen-bond to every phosphate group and to every base edge. Even though the pattern of backbone conformations is non-identical in the two structures, much of the spine is conserved in these structures, with a common feature of seven deeply embedded water molecules arranged in a quasi-linear one dimensional array, as seen especially clearly in Figure 4A (waters colored in cyan). These all have low mobilities as indicated by their low *B* factors (Figures 4B and 5B).

6JKN is the only quadruplex structure studied here that has two narrow grooves. Well-structured water spines span both those grooves connecting at the bottom side of the structure (side with two lateral loops). Quadruplex folding of 6JKN can be hypothesized to initiate with a non-canonical duplex hairpin (caped by T10–T11–A12 loop) maintained by the highly structured water network working in concert with the non-canonical hydrogen bonding between bases. Subsequent folding of the hairpin will lead to the formation of the quadruplex with the aid of the Hoogsteen hydrogen bonding between guanines.

These patterns of ordered water spines are fundamentally distinct from the well-characterized water spine in A/T-rich regions of duplex DNAs (7–14). In these structures, primary-sphere waters inter-strand hydrogen-bond to base-pair edges. These waters do not directly contact each other but are connected by intervening second-sphere waters. There are no water-phosphate group contacts, rather water–O4′ contacts. Even though a narrow quadruplex groove, for example in 6JKN and 6XT7, has at first sight the appearance of a duplex groove, with base-pair edges forming the floor and phosphodiester groups the walls of the groove, in recognition terms it is very distinct with only one base of the G:G base pair presenting hydrogen bond potential (with N2 and N3 atoms). The other G base presents a hydrophobic C8 atom at the groove floor, forcing water molecules to one side of the groove where they contact either phosphate or O4’ atoms.

The existence of structured water molecules with long residence times in quadruplex grooves have been previously suggested on the basis of molecular dynamics simulations of solvated quadruplexes (36,37), although the detailed predictions of the arrangements are not in accord with the observations described here. The water molecules in the 1JPQ structure (38) have been previously noted (39) to be in accord with simulation studies and to form a spine. The water arrangement in the grooves of the simple tetramolecular parallel-stranded quadruplex formed by the sequence TGGGGT (PDB id 352D at 0.95 Å) also shows connected water networks in several grooves (40). This structure differs fundamentally from the unimolecular parallel quadruplexes analyzed here in that it is a loop-free structure and thus its hydration patterns have only limited relevance to those in genomic parallel quadruplexes. The importance of hydration in stabilizing quadruplexes, notably the human telomeric quadruplex, has been highlighted by biophysical and thermodynamic studies (41). The observation here of stable extended water spines in anti-parallel and hybrid quadruplex structures but not in parallel ones suggests that the transition for the human telomeric quadruplexes from hybrid and anti-parallel arrangements to the parallel topology in more crowded conditions (41–43) is driven by a loss of the stable water spine structure. The finding that water molecules can form extended and stable structures in quadruplex grooves suggests the possibility of designing specialized groove-binding small molecules capable of displacing a water spine, and thus displaying selectivity for one topology over another. Water structure in the grooves of the highly distinctive topology shown by left-handed quadruplexes (44,45) has not been discussed here. The left-handed quadruplex formed by the sequence T(GGT)_4_TGT(GGT)_3_GTT (structure PDB id 4U5M) is at high resolution (1.5 Å) and has a single dominant zig-zag shaped narrow groove that is almost parallel to the G-quartet planes and winds around ca 300° of the exterior of the G-quartet core (44). The complex pattern of hydration in this groove is very distinct from those observed in the right-handed quadruplexes described here and will the subject of further comparative study especially once other left-handed structures become available.

## Supplementary Material

gkaa1177_Supplemental_File

## References

[1] Biedermannová L., Schneider B. Hydration of proteins and nucleic acids: advances in experiment and theory. a review. Biochim. Biophys. Acta. 2016; 1860:1821–1835. 27241846 10.1016/j.bbagen.2016.05.036

[2] Laage D., Elsaesser T., Hynes J.T. Water dynamics in the hydration shells of biomolecules. Chem. Rev. 2017; 117:10694–10725. 28248491 10.1021/acs.chemrev.6b00765 PMC5571470

[3] Berman H.M. Hydration of DNA. Curr. Opin. Struct. Biol. 1991; 1:423–427.

[4] Fuller W., Forsyth T., Mahendrasingam A. Water–DNA interactions as studied by X–ray and neutron fibre diffraction. Phil. Trans. Roy. Soc. Lond. B. 2004; 359:1237–1248. 15306379 10.1098/rstb.2004.1501 PMC1693411

[5] Franklin R.E., Gosling R.G. The structure of sodium thymonucleate fibres. I. The influence of water content. Acta Crystallogr. 1953; 6:673–677.

[6] Watson J.D., Crick F.H.C. Molecular structure of nucleic acids: a structure for deoxyribose nucleic acid. Nature. 1953; 171:737–738. 13054692 10.1038/171737a0

[7] Minasov G., Tereshko V., Egli M. Atomic-resolution crystal structures of B-DNA reveal specific influences of divalent metal ions on conformation and packing. J. Mol. Biol. 1999; 291:83–99. 10438608 10.1006/jmbi.1999.2934

[8] Vlieghe D., Turkenburg J.P., Van Meervelt L. B-DNA at atomic resolution reveals extended hydration patterns. Acta Crystallogr. D. 1999; 55:1495–1502. 10489444 10.1107/s0907444999007933

[9] Soler-López M., Malinina L., Liu J., Huynh-Dinh T., Subirana J.A. Water and ions in a high resolution structure of B-DNA. J. Biol. Chem. 1999; 274:23683–23686. 10446123 10.1074/jbc.274.34.23683

[10] Edwards K.J., Brown D.G., Spink N., Skelly J.V., Neidle S. An examination of propeller twist and minor-groove water structure at 2.2Å resolution. J. Mol. Biol. 1992; 226:1161–1173. 1518049 10.1016/0022-2836(92)91059-x

[11] Woods K.K., Maehigashi T., Howerton S.B., Sines C.C., Tannenbaum S., Williams L.D. High-resolution structure of an extended A-tract: [d(CGCAAATTTGCG)]_2_. J. Am. Chem. Soc. 2004; 126:15330–15331. 15563130 10.1021/ja045207x

[12] Arai S., Chatake T., Ohhara T., Kurihara K., Tanaka I., Suzuki N., Fujimoto Z., Mizuno H., Niimura N. Complicated water orientations in the minor groove of the B-DNA decamer d(CCATTAATGG)_2_ observed by neutron diffraction measurements. Nucleic Acids Res. 2005; 33:3017–3024. 15914673 10.1093/nar/gki616 PMC1140084

[13] Kopka M.L., Fratini A.V., Drew H.R., Dickerson R.E. Ordered water structure around a B-DNA dodecamer: A quantitative study. J. Mol. Biol. 1983; 163:129–146. 6834428 10.1016/0022-2836(83)90033-5

[14] Johansson E., Parkinson G., Neidle S. A new crystal form for the dodecamer C-G-C-G-A-A-T-T-C-G-C-G: symmetry effects on sequence-dependent DNA structure. J. Mol. Biol. 2000; 300:551–561. 10884351 10.1006/jmbi.2000.3907

[15] Liepinsh E., Leupin W., Otting G. Hydration of DNA in aqueous solution: NMR evidence for a kinetic destabilization of the minor groove hydration of d-(TTAA)_2_ versus d-(AATT)_2_ segments. Nucleic Acids Res. 1994; 22:2249–2254. 8036152 10.1093/nar/22.12.2249 PMC523681

[16] McDermott M.L., Vanselous H., Corcelli S.A., Petersen P.B. DNA’s chiral spine of hydration. ACS Cent. Sci. 2017; 3:708–714. 28776012 10.1021/acscentsci.7b00100 PMC5532714

[17] Woda J., Schneider B., Patel K., Mistry K., Berman H.M. An analysis of the relationship between hydration and protein-DNA interactions. Biophys. J. 1998; 75:2170–2177. 9788911 10.1016/S0006-3495(98)77660-X PMC1299890

[18] Jayaram B., Jain T. The role of water in protein-DNA recognition. Annu. Rev. Biophys. Biomol. Struct. 2004; 33:343–361. 15139817 10.1146/annurev.biophys.33.110502.140414

[19] Wei W., Luo J., Waldispühl J., Moitessier N. Predicting positions of bridging water molecules in nucleic acid–ligand complexes. J. Chem. Inf. Model. 2019; 59:2941–2951. 30998377 10.1021/acs.jcim.9b00163

[20] Neidle S., Berman H.M., Shieh H.S. Highly structured water network in crystals of a deoxydinucleoside–drug complex. Nature. 1980; 288:129–133. 7432511 10.1038/288129a0

[21] Nguyen B., Neidle S., Wilson W.D. A role for water molecules in DNA−ligand minor groove recognition. Acc. Chem. Res. 2009; 42:11–21. 18798655 10.1021/ar800016q PMC2668807

[22] Wei D., Wilson W.D., Neidle S. Small-molecule binding to the DNA minor groove is mediated by a conserved water cluster. J. Am. Chem. Soc. 2013; 135:1369–1377. 23276263 10.1021/ja308952y PMC3580125

[23] Burge S., Parkinson G.N., Hazel P., Todd A.K., Neidle S. Quadruplex DNA: sequence, topology and structure. Nucleic Acids Res. 2006; 34:5402–5415. 17012276 10.1093/nar/gkl655 PMC1636468

[24] Spiegel J., Adhikari S., Balasubramanian S. The structure and function of DNA G-quadruplexes. Trends Chem. 2:123–136. 32923997 10.1016/j.trechm.2019.07.002 PMC7472594

[25] Zyner K.G., Mulhearn D.S., Adhikari S., Martínez Cuesta S., Di Antonio M., Erard N., Hannon G.J., Tannahill D., Balasubramanian S. Genetic interactions of G-quadruplexes in humans. eLife. 2019; 8:e46793. 31287417 10.7554/eLife.46793 PMC6615864

[26] Di Antonio M., Ponjavic A., Radzevičius A., Ranasinghe R.T., Catalano M., Zhang X., Shen J., Needham L.-M., Lee S.F., Klenerman D., Balasubramanian S. Single-molecule visualization of DNA G-quadruplex formation in live cells. Nat. Chem. 2020; 12:832–837. 32690897 10.1038/s41557-020-0506-4 PMC7610488

[27] Asamitsu S., Bando T., Sugiyama H. Ligand design to acquire specificity to intended G-quadruplex structures. Chemistry. 2019; 25:417–430. 30051593 10.1002/chem.201802691

[28] Neidle S. Quadruplex nucleic acids as novel therapeutic targets. J. Med. Chem. 2016; 59:5987–6011. 26840940 10.1021/acs.jmedchem.5b01835

[29] Burley S.K., Berman H.M., Bhikadiya C., Bi C., Chen L., Di Costanzo L., Christie C., Dalenberg K., Duarte J.M., Dutta S. et al. RCSB Protein Data Bank: biological macromolecular structures enabling research and education in fundamental biology, biomedicine, biotechnology and energy. Nucleic Acids Res. 2019; 47:D464–D474. 30357411 10.1093/nar/gky1004 PMC6324064

[30] Clark G.R., Squire C.J., Baker L.J., Martin R.F., White J. Intermolecular interactions and water structure in a condensed phase B-DNA crystal. Nucleic Acids Res. 2000; 28:1259–1265. 10666471 10.1093/nar/28.5.1259 PMC102594

[31] Goddard T.D., Huang C.C., Meng E.C., Pettersen E.F., Couch G.S., Morris J.H., Ferrin T.E. UCSF chimeraX: meeting modern challenges in visualization and analysis: UCSF chimeraX visualization system. Protein Sci. 2018; 27:14–25. 28710774 10.1002/pro.3235 PMC5734306

[32] Emsley P., Lohkamp B., Scott W.G., Cowtan K. Features and development of Coot. Acta Crystallogr. D. 2010; 66:486–501. 20383002 10.1107/S0907444910007493 PMC2852313

[33] Winn M.D., Ballard C.C., Cowtan K.D., Dodson E.J., Emsley P., Evans P.R., Keegan R.M., Krissinel E.B., Leslie A.G.W., McCoy A. et al. Overview of the CCP4 suite and current developments. Acta Crystallogr. D. 2011; 67:235–242. 21460441 10.1107/S0907444910045749 PMC3069738

[34] Potterton E., Briggs P., Turkenburg M., Dodson E. A graphical user interface to the CCP4 program suite. Acta Crystallogr. D. 2003; 59:1131–1137. 12832755 10.1107/s0907444903008126

[35] Collie G.W., Campbell N.H., Neidle S. Loop flexibility in human telomeric quadruplex small-molecule complexes. Nucleic Acids Res. 2015; 43:4785–4799. 25940631 10.1093/nar/gkv427 PMC4446451

[36] Strahan G.D., Keniry M.A., Shafer R.H. NMR structure refinement and dynamics of the K^+^-[d(G_3_T_4_G_3_)]_2_ quadruplex via Particle Mesh Ewald molecular dynamics simulations. Biophysical J. 1998; 75:968–981. 10.1016/S0006-3495(98)77585-X PMC1299770 9675197

[37] Sheu S.-Y., Liu Y.-C., Zhou J.-K., Schlag E.W., Yang D.-Y. Surface topography effects of globular biomolecules on hydration water. J. Phys. Chem. B. 2019; 123:6917–6932. 31282162 10.1021/acs.jpcb.9b03734

[38] Haider S., Parkinson G.N., Neidle S. Crystal structure of the potassium form of an *Oxytricha nova* G-quadruplex. J. Mol. Biol. 2002; 320:189–200. 12079378 10.1016/S0022-2836(02)00428-X

[39] Giambaşu G.M., Case D.A., York D.M. Predicting site-binding modes of ions and water to nucleic acids using molecular solvation theory. J. Am. Chem. Soc. 2019; 141:2435–2445. 30632365 10.1021/jacs.8b11474 PMC6574206

[40] Phillips K., Dauter Z., Murchie A.I., Lilley D.M., Luisi B. The crystal structure of a parallel-stranded guanine tetraplex at 0.95 Å resolution. J. Mol. Biol. 1997; 273:171–182. 9367755 10.1006/jmbi.1997.1292

[41] Miller M.C., Buscaglia R., Chaires J.B., Lane A.N., Trent J.O. Hydration Is a major determinant of the G-quadruplex stability and conformation of the human telomere 3′ sequence of d(AG_3_(TTAG_3_)_3_). J. Am. Chem. Soc. 2010; 132:17105–17107. 21087016 10.1021/ja105259m

[42] Petraccone L., Pagano B., Giancola C. Studying the effect of crowding and dehydration on DNA G-quadruplexes. Methods. 2012; 57:76–83. 22406490 10.1016/j.ymeth.2012.02.011

[43] Yu H., Gu X., Nakano S., Miyoshi D., Sugimoto N. Beads-on-a-string structure of long telomeric DNAs under molecular crowding conditions. J. Am. Chem. Soc. 2012; 134:20060–20069. 22934853 10.1021/ja305384c

[44] Chung W.J., Heddi B., Schmitt E., Lim K.W., Mechulam Y. and, Phan A.T. Structure of a left-handed DNA G-quadruplex. Proc. Natl. Acad. Sci. USA. 2015; 112:2729–2733. 25695967 10.1073/pnas.1418718112 PMC4352798

[45] Winnerdy F.R., Bakalar B., Maity A., Vandana J.J., Mechulam Y., Schmitt E., Phan A.T. NMR solution and X-ray crystal structures of a DNA molecule containing both right- and left-handed parallel-stranded G-quadruplexes. Nucleic Acids Res. 2019; 47:8272–8281. 31216034 10.1093/nar/gkz349 PMC6735952

[46] Ou A., Schmidberger J.W., Wilson K.A., Evans C.W., Hargreaves J.A., Grigg M., O’Mara M.L., Iyer K.S., Bond C.S., Smith N.M. High resolution crystal structure of a KRAS promoter G-quadruplex reveals a dimer with extensive poly-A π-stacking interactions for small-molecule recognition. Nucleic Acids Res. 2020; 48:5766–5776. 32313953 10.1093/nar/gkaa262 PMC7261167

[47] Geng Y., Liu C., Zhou B., Cai Q., Miao H., Shi X., Xu N., You Y., Fung C.P., Din R.U. et al. The crystal structure of an antiparallel chair-type G-quadruplex formed by bromo-substituted human telomeric DNA. Nucleic Acids Res. 2019; 47:5395–5404. 30957851 10.1093/nar/gkz221 PMC6547763

[48] Parkinson G.N., Lee M.P.H., Neidle S. Crystal structure of parallel quadruplexes from human telomeric DNA. Nature. 2002; 417:876–880. 12050675 10.1038/nature755

[49] Nuthanakanti A., Ahmed I., Khatik S.Y., Saikrishnan K., Srivatsan S.G. Probing G-quadruplex topologies and recognition concurrently in real time and 3D using a dual-app nucleoside probe. Nucleic Acids Res. 2019; 47:6059–6072. 31106340 10.1093/nar/gkz419 PMC6614846

[50] Neidle S. Principles of Nucleic Acid Structure. 2008; NY Academic Press 20–37.

