# Supplementary material for: Water spines and networks in G-quadruplex structures
Source: Nucleic Acids Res. 2020 Dec 8;49(1):519–28. doi: 10.1093/nar/gkaa1177 (PMC7797044; doi:10.1093/nar/gkaa1177)
Supplement: gkaa1177_Supplemental_File [file gkaa1177_supplemental_file.docx]

**Supplementary Data**

**Table S1.** Number of water molecules interacting with a specific feature of the DNA quadruplex structures or with the secondary sphere and other water molecules.

| Contacts | 7KLP | 6N65 | 6XT7 | 6JKN | 1JPQ |
| --- | --- | --- | --- | --- | --- |
| Total waters | 90 | 64 | 286 | 119 | 131 |
| Primary contacts | 68 | 53 | 64 ± 5* | 98 | 68 |
| PO4 | 42 | 36 | 37 ± 3* | 71 | 47 |
| Water | 61 | 35 | 55 ± 7* | 81 | 95 |
| O4’ | 6 | 14 | 6 ± 1* | 3 | 5 |
| N2 | 9 | 20 | 11 ± 2* | 7 | 12 |
| N3 | 7 | 16 | 7.5 ± 0.5* | 6 | 7 |

*per quadruplex; numbers represent an average for four quadruplexes in the asymmetric unit with the confidence interval of 95%.

**Table S2.** Number of water molecules involved in two-way interactions.

| Contacts | 7KLP | 6N65 | 6XT7* | 6JKN | 1JPQ |
| --- | --- | --- | --- | --- | --- |
| PO4-HOH | 26 | 16 | 31 | 43 | 32 |
| PO4-O4' | 1 | 8 | 1 | 2 | 0 |
| PO4-N2 | 1 | 12 | 3 | 2 | 4 |
| PO4-N3 | 0 | 10 | 0 | 0 | 0 |
| HOH-O4’ | 7 | 8 | 4 | 2 | 5 |
| HOH-N2 | 10 | 14 | 10 | 6 | 9 |
| HOH-N3 | 6 | 8 | 7 | 5 | 5 |
| O4’-N2 | 6 | 12 | 2 | 0 | 3 |
| O4’-N3 | 4 | 6 | 1 | 0 | 1 |
| N2-N3 | 2 | 8 | 1 | 1 | 0 |

* numbers represent an average for four quadruplexes in the asymmetric unit

**Table S3.** Number of water molecules involved in three-way interactions.

| Contacts | 7KLP | 6N65 | 6XT7* | 6JKN | 1JPQ |
| --- | --- | --- | --- | --- | --- |
| PO4-HOH-O4’ | 1 | 4 | 0 | 1 | 0 |
| PO4-HOH-N2 | 1 | 8 | 2.75 | 1 | 3 |
| PO4-HOH-N3 | 0 | 3 | 0 | 0 | 0 |
| PO4-O4’-N2 | 1 | 8 | 0 | 0 | 0 |
| PO4-O4’-N3 | 0 | 2 | 0 | 0 | 0 |
| PO4-N2-N3 | 0 | 4 | 0 | 0 | 0 |
| HOH-O4’-N2 | 6 | 6 | 2.25 | 0 | 3 |
| HOH-O4’-N3 | 3 | 4 | 1 | 0 | 1 |
| HOH-N2-N3 | 2 | 6 | 0.5 | 1 | 0 |
| O4’-N2-N3 | 2 | 5 | 0.5 | 0 | 0 |

* numbers represent an average for four quadruplexes in the asymmetric unit

**Table S4.** Number of water molecules involved in four-way interactions.

| Contacts | 7KLP | 6N65 | 6XT7 | 6JKN | 1JPQ |
| --- | --- | --- | --- | --- | --- |
| PO4-HOH-O4’-N2 | 1 | 4 | 0 | 0 | 0 |
| PO4-HOH-O4’-N3 | 0 | 1 | 0 | 0 | 0 |
| PO4-HOH-N2-N3 | 0 | 3 | 0 | 0 | 0 |
| PO4-O4’-N2-N3 | 0 | 2 | 0 | 0 | 0 |
| HOH-O4’-N2-N3 | 2 | 3 | 0.5 | 0 | 0 |

Note, only 6N65 has one water molecule interacting with all five groups, PO4-O4’-N2-N3-HOH.

**Figure S1.** The effects of *anti* vs *syn* glycosidic angles on the position and orientation of O4’ atoms and phosphate groups (shown is a part of structure 6XT7).

**
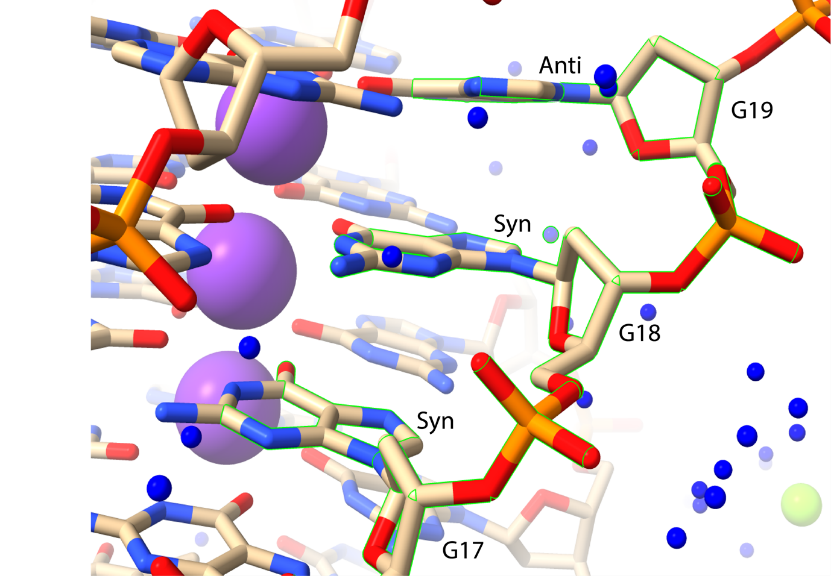
**
